# Medicinal Plants against Viral Infections: A Review of Metabolomics Evidence for the Antiviral Properties and Potentials in Plant Sources

**DOI:** 10.3390/v16020218

**Published:** 2024-01-31

**Authors:** Wilson Bamise Adeosun, Du Toit Loots

**Affiliations:** Human Metabolomics, North-West University, Private Bag X6001, Box 269, Potchefstroom 2531, South Africa; dutoit.loots@nwu.ac.za

**Keywords:** viral infections, antiviral activity, medicinal plants, metabolomics

## Abstract

Most plants have developed unique mechanisms to cope with harsh environmental conditions to compensate for their lack of mobility. A key part of their coping mechanisms is the synthesis of secondary metabolites. In addition to their role in plants’ defense against pathogens, they also possess therapeutic properties against diseases, and their use by humans predates written history. Viruses are a unique class of submicroscopic agents, incapable of independent existence outside a living host. Pathogenic viruses continue to pose a significant threat to global health, leading to innumerable fatalities on a yearly basis. The use of medicinal plants as a natural source of antiviral agents has been widely reported in literature in the past decades. Metabolomics is a powerful research tool for the identification of plant metabolites with antiviral potentials. It can be used to isolate compounds with antiviral capacities in plants and study the biosynthetic pathways involved in viral disease progression. This review discusses the use of medicinal plants as antiviral agents, with a special focus on the metabolomics evidence supporting their efficacy. Suggestions are made for the optimization of various metabolomics methods of characterizing the bioactive compounds in plants and subsequently understanding the mechanisms of their operation.

## 1. Introduction

The continuous emergence of viral diseases as a major challengeto global health warrants an intensified effort from a combined team of researchers and healthcare professionals. Continuous or regularly occurring pathogenic viral infections of pandemic proportions account for millions of deaths annually [1]. Although the development of conventional antiviral drugs is a significant stride toward curbing this trend, in the last decade, limitations have become increasingly conspicuous. The limitations include issues such as drug resistance [2], a narrow spectrum of efficacy [3], costs [4], and adverse side effects [5]. This has led to a resurgence of interest in the exploration of alternative treatment modalities from health stakeholders. Numerous chemical compounds found in natural sources are exclusive to plants, microbes, and marine life [6]. They provide important leads for drug discoveries and can potentially contribute to antiviral treatment/drug development [7,8,9]. Compounds derived from natural sources play a crucial role in drug discovery and the development of new antiviral treatments [7,10]. Medicinal plants have emerged as a promising frontier in the search for innovative antiviral therapies [11]. 

Throughout history, different cultures have sought healing in the potency of plants for the alleviation of pain and diseases. Their use in traditional medicine systems globally underscores their potential as a source of valuable antiviral compounds. 

Plants respond to biotic and abiotic stress by synthesizing a large array of secondary metabolites with complex chemical combinations [12,13,14]. Although numerous studies have reported the activities and potentials of plants against viruses [15,16], it is important to validate traditional remedies through clinical trials [17,18]. 

The application of advanced analytical techniques to modern scientific research presents an opportunity for the exploration of plant-based compounds for their therapeutic effects. Metabolomics has emerged as an indispensable tool for the identification of different classes of antiviral secondary metabolites from plants over the last decade. These include various flavonoids, terpenoids, alkaloids, and polyphenols, each of which exhibit various therapeutic effects during different stages of the viral cycle (e.g., viral attachment, entry, viral replication, and release) [19]. These identified antiviral plant metabolites account for about a quarter of all drugs developed and used today [20,21].

The interplay between medicinal plants, viral infections, and metabolomics offers a multifaceted view of the potentials inherent in plants for the development of new antiviral therapeutics. This review highlights the role of metabolomics in the discovery and development of new antiviral bioactive compounds from medicinal plants. It also identifies the obvious gaps in current knowledge and offers comprehensive suggestions to future research directions. 

## 2. Overview of Viral Infections

Viruses have been part of life since time immemorial. They are the smallest known agents of human infections, with a diameter that ranges between 20–200 nanometers [22]. Viruses are ubiquitous and can be found in animals, plants, humans, and other living organisms. All viruses are referred to as obligate intracellular pathogens since they cannot complete their life cycle without a living host [23]. Many of them are benign, non-pathogenic, and can even save lives. Bacteriophages, for instance, have been used as natural predators for therapeutic purposes, either exclusively in cases of failed conventional antibiotic therapy or in conjunction with antibiotics with satisfactory outcomes [24,25,26]. Some, however, do contribute to a considerable number of infections in humans [27,28]. Viral infections present a significant public health concern. Over the last few decades, many new viruses have emerged, with a significant number of them having deleterious effects on human health [29]. There are reportedly 26 virus families implicated in human diseases, with each exhibiting a different genomic structure, physiochemical properties, molecular processes, and morphology [30].

Viral infections are diverse and include: (1) sexually transmitted infections, including hepatitis B, HIV, herpes simplex virus (HSV), and human papillomavirus (HPV); (2) gastrointestinal infections, which lead to gastroenteritis caused by noroviruses, rotaviruses, adenoviruses, and sapoviruses; (3) zoonotic infections, caused by viruses that can be hosted by both animals and humans, e.g., Ebola, rabies, and hantaviruses; (4) hepatic infections, which can result in hepatitis, e.g., hepatitis A, B, C, D, and E and others including yellow fever and the Epstein-Barr virus; (5) Respiratory infections such as the common cold (caused by rhinoviruses), flu (caused by influenza viruses), COVID-19 (caused by the SARS-CoV-2 virus), and the respiratory syncytial virus (RSV) [30,31,32].

The most common of these viral diseases are those that affect the respiratory system [33]. A collaboration between researchers from several institutions and countries reported that respiratory diseases were the third leading cause of death worldwide between the years 1990 and 2019, beneath only cancer and cardiovascular diseases [34]. The influenza virus, which is the causative agent for the acute respiratory infection seasonal flu, remains one of the biggest threats to public health according to the World Health Organization (WHO). The organization gave an estimate of 290,000–650,000 flu-related deaths annually [35]. SARS-CoV-2 accounted for at least 14.9 million deaths directly or indirectly at the height of COVID-19 pandemic (between the years 2020 and 2021) according to WHO [36]. 

Viral infections are risk factors for other medical conditions, since they have the potential to weaken the immune system and induce an inflammatory response from the host cell [29,37]. Recent studies confirmed that oncogenic viruses implicated in the development of some cancers account for about 10% of all global cancer burden [38], and may also be responsible for long-term persistent infections [39]. Viral infections can also result in secondary bacterial infections, a condition in which infected patients are predisposed to health complications from bacterial sources due to a weakened immune system [40,41,42]. In the past, cardiovascular diseases have also been linked to viral infections [43]. In 1932, [44] reported that the peak period of an influenza pandemic in the United States was in direct proportion to an increase in heart diseases. Also, in the wake of the COVID-19 pandemic, there were many reports of heightened cases of cardiovascular diseases linked to the SARS-CoV-2 virus [45,46].

The unique nature of viruses and their ability to rapidly mutate, leading to an emerging pathogenesis and drug resistance, makes the current range of therapeutic and prophylactic options available for treating viral infections increasingly smaller [2,47,48,49]. Plants have been used for centuries as part of traditional medicine in the treatment of a variety of diseases, including various viral infections. Since the first attempt to screen over 200 plants for anti-influenza activity some seven decades ago [50], many additional studies have shown the vast potential of an enormous array of various medicinal plants across diverse geographical locations. Plants display antiviral activities and potentials for antiviral drug development, which can be used as a standalone treatment or as complementary therapeutic agents to conventional antiviral medicines [51,52,53,54,55,56]. 

Metabolomics, which is the study of all metabolites present in a biological system at a given time, has become a valuable tool for the identification and quantification of possible new therapeutic compounds from plants. A combination of techniques including Nuclear Magnetic Resonance (NMR) and advanced hyphenated mass spectrometry, are now central to metabolomics studies and are routinely applied to new biomarker identification. 

## 3. A Brief History of the Use of Medicinal Plants against Viral Infections

Medicinal plant use against a variety of viral infections across various cultures dates to the dawn of human civilization. Traditional Chinese medicine, the Eber papyrus of Ancient Egypt, and the Ayurveda of India are among the oldest cultures practicing medicinal plant use with well documented manuscripts available for such [57,58,59]. Ancient traditional Chinese medicine has a history of about 3000 years or more. Writings based on this practice describing the use of plants for healing purposes are among the oldest medical writings of any culture [60]. Examples of plants used to treat viral infections in ancient China include ephedra (*Ephedra sinica*) for the treatment of the common cold [61], *Andrographis paniculata* for treating coughs, colds, and influenza [62], *Camellia sinensis* (green tea) which has confirmed efficacy against herpes, hepatitis B and C, and Epstein-Barr viruses [63,64,65]. The Egyptian papyrus contains descriptions of plant and natural product preparations against a plethora of diseases, including various viral infections [66]. One of the plants discussed is garlic (*Allium sativum*) [67], used for the treatment of respiratory catarrh, influenza, and recurring colds [68]. Echinacea (*Echinacea purpurea*), also described in the papyrus, has been reported to have efficacy against respiratory viral infections [69]. Ayurveda, a natural system of medicine with historical roots in India dating back three millennia, describes the plants *Aegle marmelos*, *Ficus religiosa*, and *Azadirachta indica,* among others, to have efficacy against a variety of viruses [57,70,71,72]. European herbal medicine and traditional African medicine also have long histories of using plants to combat various illnesses, including viral infections. Elderberry (*Sambucus nigra*), for instance, has been used in Europe for many years as a remedy for colds and flu [73], while plants like *Sutherlandia frutescens* and *Artemisia afra*, native to Africa, have reported potency against various viral infections [74,75]. The last few decades have seen a resurgence in the use of medicinal plants as an alternative source of treatments for various viral infections [76]. This is due in part to the rise in the prevalence of viral infections and growing concern about antibiotic resistance [2,77]. 

## 4. Mechanism of Actions of Antiviral Secondary Metabolites in Medicinal Plants

Plants’ secondary metabolites are organic compounds that are not directly involved in the growth, development, or reproduction of the plants. They are produced as a survival strategy against adverse conditions in the surrounding environment and to carry out important physiological tasks [12]. There are various criteria used in determining the classification of secondary metabolites in plants. and include chemical structure, composition of constituent elements, and how soluble they are in water or organic solvents. The most commonly accepted criterion however is their biosynthetic pathway [78]. Based on these pathways, three classes of secondary metabolites have been identified in medicinal plants: (1) alkaloids, (2) terpenoids and (3) phenolic compounds [79]. Each exhibits different phytochemical constituents and pharmacological effects against various viral agents [78,80]. Commonly reported mechanisms of action of plant-derived secondary metabolites against viruses include: (1) virus entry attachment [81,82], (2) inhibition of viral replication [19,80], (3) protein synthesis inhibition [83,84,85], (4) modulation of the host’s immune system [86], (5) modulation of cellular signaling pathways [87], and (6) direct virucidal activity [87,88].

### 4.1. Phenolics

Phenolic compounds are a diverse group of plant-derived organic molecules structurally characterized by at least one phenol group. They are the most widely distributed secondary metabolites in plants [89] and are synthesized as an adaptive response to unfavorable conditions [90]. Phenols are commonly found in all plant organs and are rich constituents of fruits, vegetables, beverages, cereals, and legumes [91]. Some well-known phenolic compounds with reported antiviral activities include flavonoids, tannins, and phenolic acids [92,93,94]. The specific mechanism of action of phenols against viruses depends on the type of phenolic compound and the virus targeted. 

Phenols are well-known to inhibit viral infection of a target host cell in a variety of ways [95,96,97,98,99], including the disruption of the virus lipid bilayer envelopes [100,101] and preventing viral attachment to the host cell [97,102]. Furthermore, the inhibition of various viral protein activities results in the disruption of the viral life cycle, which ultimately prevents the multiplication and release of the virus [85,102,103].

Various phenols have also been shown to interfere with viral replication by binding to and subsequently inactivating various viral proteins or enzymes, and subsequently halt disease progression in the host cells [102,104,105]. Specific examples of compounds reported to interfere with viral replication include the tannin epigallocatechin-3-gallate (EGCG), a catechin derived from green tea [106]. EGCG demonstrates inhibition of the M2 protein of the influenza A virus by increasing IFN-λ2 expression in human lung epithelial BEAS-2B cells through the p38 mitogen-activated protein kinase signaling pathway [107]. EGCG has also been reported to inhibit the binding of HIV-1 envelope glycoprotein gp120 to the glycoprotein CD4 receptor found on an immune cell’s surface [108,109]. Four flavonoids, namely quercetin, baicalein, myricetin, and quercetagetin, have also previously been reported to inhibit HIV-1 enzyme reverse transcriptase and cellular DNA and RNA polymerases [110]. 

Sodium ferulate, another phenolic acid found abundantly in plants [111], inhibits the replication of influenza viruses via the activation of the toll-like receptors TLR7 and TLR9, and subsequently promotes transcription factor IRF7’s translocation into the host nucleus and the production of type-1 interferons [112]. Chicoric acid such as (caffeic acid derivative) and a number of dicaffeoylquinic acids have been reported to interfere with the enzyme integrase of HIV, an enzyme that is crucial to the integration of viral DNA into the host genome [113]. Gallic acid has also been shown to possess anti-HSV-2 properties, primarily through the inhibition of the virus’s attachment to the host cell, subsequently limiting the spread of the virus [114]. Furthermore, multiple antiviral defense mechanisms, including the inhibition of viral replication and cell to cell movement have been reportedly activated by salicylic acid [115]. The immunomodulatory effects of various phenolic compounds have also been previously shown to activate the prouction of various host cytokines and chemokines [116,117]. Lastly, a number of plant-derived phenolic compounds have been shown to induce apoptosis in an infected host, limiting the spread of viral infections [102,118,119,120]. 

**Figure 1:**
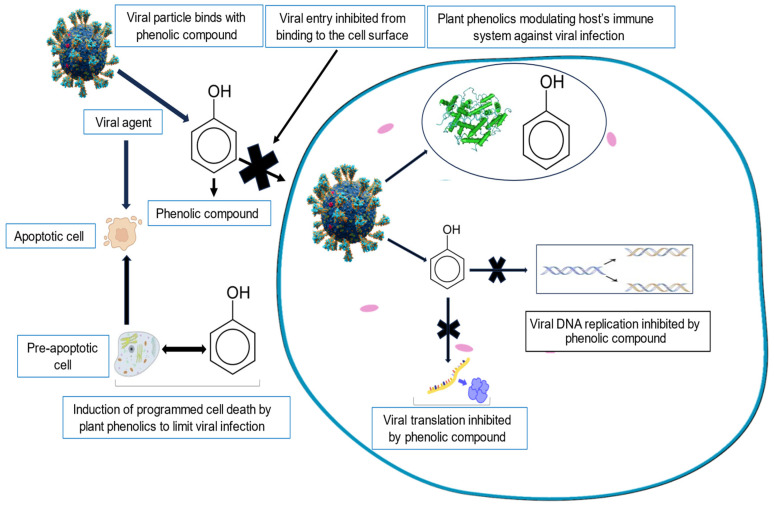
A schematic representation showing the mechanisms of action of the antiviral properties of phenolic compounds.

Figure 1 schematically summarizes the antiretroviral effects of plant-derived phenolic compounds. 

### 4.2. Alkaloids

Alkaloids, primarily but not exclusively present in plants, are a class of naturally occurring organic compounds containing at least one nitrogen atom. They can be found in many plant structures and have been reported to have pharmacological effects against various microbial diseases and viruses. Alkaloids have in particular been highlighted for their broad-spectrum activities against both DNA and RNA viruses [121,122]. Due to their importance, they have also been identified as the largest class of plants’ secondary metabolites investigated to date [122]. The most well-known plant alkaloids found in nature include cocaine, morphine, and quinine. 

Alkaloids have been identified as important inhibitors of the flow of genetic information (from the DNA/RNA viral particle to protein synthesis) necessary to ensure the lifecycle of the virus. The antiviral activities of alkaloids, as proven by experimental evidence, primarily involve the inhibition of: (1) DNA and RNA replication, (2) RNA translation, (3) protein synthesis, (4) DNA intercalation, (5) enzymatic activities, (6) the translocation of the ribonucleoprotein complex, (7) DNA synthesis, and (8) protein synthesis. Many studies have reported the use of alkaloids for the treatment and prevention of viral infections [122,123,124]. Alkaloids can also regulate human immune mechanisms toward viral resistance by mediating the humoral immune response [125]. 

**Figure 2:**
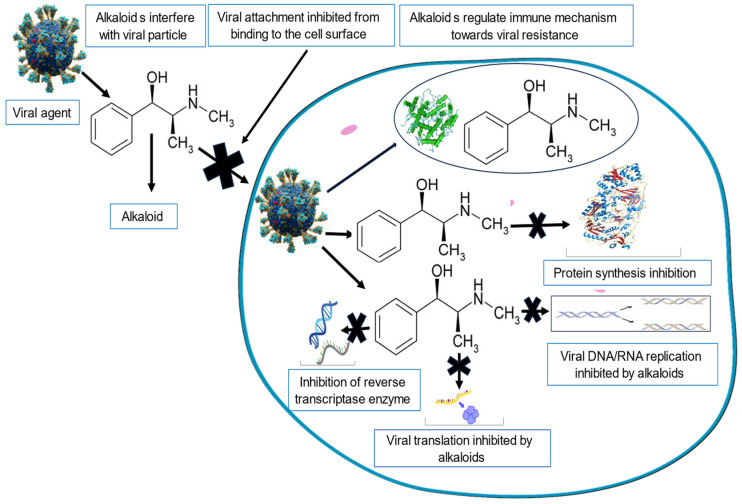
A schematic diagram showing the mechanism of the antiviral activities of alkaloids.

Figure 2 below is a schematic representation of the antiviral effects of alkaloids.

### 4.3. Terpenoids

Terpenoids are a large group of diverse organic products that are ubiquitous in nature. They exist in six categories namely: hemiterpenes, monoterpenes, sesqui-terpenes, diterpenes, sesterpenes, triterpenes, and tetra-terpenoids [126]. As an essential component of all living cells, they are products of both primary and secondary cellular metabolism [127]. Terpenoids are mostly present in the leaves and fruits of higher plants, where they sometimes contribute to their vibrant colors. They are also highly volatile and combustible organic compounds [128]. As aromatic metabolites, they are largely responsible for the flavor and fragrance of plants [129]. Terpenoids are of special interest to medical chemists because of their significant pharmacological potential [130]. Common terpenoids include citral, menthol camphor, and salvinorin A.

Various terpenoids have been shown to possess promising antimicrobial and antiviral properties [131]. Those with reported antiviral properties include glycyrrhizin, an important antiviral chemical compound found in the roots of the licorice plant (Glycyrrhiza glabra) [132]. Historical sources from China [133], India [134], and parts of Europe [135] make references to the use of glycyrrhizin in the treatment of viral respiratory tract and liver infections caused by hepatitis. Ref. [136] reported the immunomodulatory activities of glycyrrhizin due to the induction of interferon gamma, and [137] described its anti-inflammatory mechanisms. Glycyrrhizin furthermore inhibits replication of severe acute respiratory syndrome associated with corona virus (SARS-Chikungunya virus (CV)) infection and prevents the adsorption and penetration of the virus [138]. Isoborneol, a monoterpene present in a variety of different essential oils, totally inhibits replication of the herpes simplex virus (HSV-1), and that at a concentration of only 0.06% [139]. Additionally, β-pinene and limonene monoterpenes present in various essential oils, showed high anti-HSV-1 activity, and function by reducing the viral infectivity by 100%, by directly interfering with free viral particles [140]. Many studies have also indicated that celastrol, a pentacyclic triterpenoid, inhibits the replication of the dengue virus [141], human immunodeficiency virus [142], and hepatitis C virus [143]. 

Ref. [144] reported that the extracted pigment of carotenoids of Natrialba sp. M6, a tetraterpene isolated from Wadi El-Natrun in Egypt, exhibited antiviral potency against Hepatitis C Virus (HCV) and HBV. The RNA and DNA polymerase enzymes responsible for the amplification of the virus in both HCV and HBV were inhibited, thereby suppressing the viruses’ replication. Molecular docking simulation was also used to predict the antiviral effects of two marine carotenoids (specifically fucoxanthin and siphonaxanthin) against SARS-CoV-2. The effects were later confirmed by in vitro studies [145].

**Figure 3:**
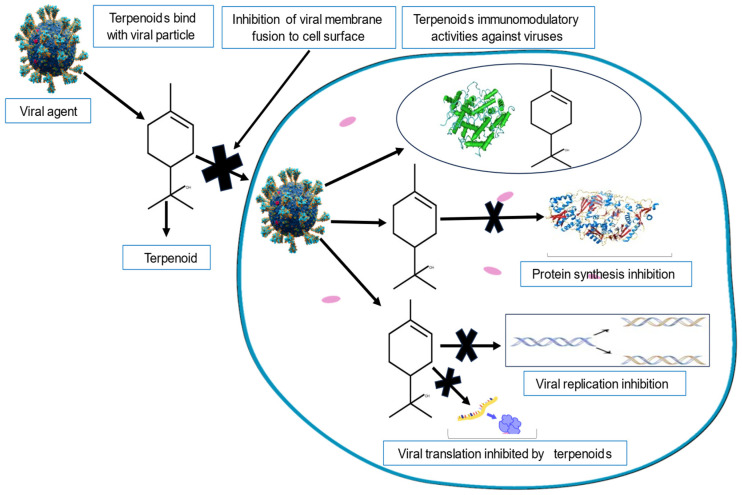
A schematic diagram showing the mechanism of the antiviral activities of terpenoids.

Figure 3 shows a schematic summary of the antiviral activities of terpenoids.

## 5. Applications of Metabolomics in Plant Antiviral Research

Metabolomics involves the comprehensive study of small molecules known as metabolites in a living system or biological sample. It can give broad insight into the identity and detailed information on the chemical fingerprints of metabolites and the metabolic processes that occur within a biological system [146]. Metabolomics is broadly categorized into two types, (1) targeted and (2) untargeted metabolomics. In the targeted approach, the goal is not the identification of all metabolites, but the quantitative measurement of a specific metabolite or metabolite group that has been previously identified and characterized. Untargeted metabolomics deals with a comprehensive identification and quantification of all detectable metabolites present in a biological sample [147]. 

Advances in metabolomic techniques now play a significant role in the discovery of antiviral compounds in plants, as evidenced by relevant recent publications. The possibilities for the comprehensive analysis of the complete set of compounds in plants using advanced analytical techniques are endless. Many studies have reported the use of metabolomics as an essential tool for the targeted screening of plant secondary metabolites [148,149]. The many advantages that metabolomics presents make it an essential and indispensable technique in current plant antiviral research. 

Arguably the most important application of metabolomics in plant antiviral research is in the identification and characterization of the plant metabolome. Metabolomics, when used for this, can provide a detailed snapshot of a plant’s metabolomic profile, with information on both the identification and quantification of the metabolites present within the metabolome. There is an incredible diversity of secondary metabolites exhibited by plants, many of which have bioactive compounds [150,151,152]. The use of metabolomics has additionally assisted in identifying a wide range of metabolites in plants across different geographical locations and seasons, including those with antiviral potential. The specific bioactive compounds responsible for the antiviral activity can then be isolated and further studied for potential therapeutic use. 

Metabolomics can also be used for mechanistic or toxicology studies of the isolated plant antiviral compounds in vivo [153,154]. Since metabolites are downstream products of cellular metabolism [153], the identification, quantification, and characterization of these compounds in vivo, in the presence of the isolated plant metabolite/drug show the metabolic pathways that are altered in response to the plant compound/drug’s antiviral activity in the culture or host. 

There are many complex metabolic interactions that occur within the plant system. Antiviral compounds can either act alone or in synergy with other compounds to carry out their activities. In studying the synergistic effect of more than one compound on microbes, stronger bioactivities have been reported using metabolomics [155,156,157,158]. The metabolomics approach is therefore promising, as a useful strategy for the study of complex interactions between plant metabolites [159,160]. 

Metabolomics has also been applied to investigating the effect of the different seasons on the chemical profiles and biological activities of plants [161,162]. Ref. [163] used metabolomics to study the influence of seasonal change on the anti-HSV1 properties of *Helichrysum aureonitens* and reported a correlation between plants harvested in spring and better antiviral activity. A targeted metabolomics study conducted to determine the effect of seasonal change on the chlorogenic acids content of *H. aureonitens* further revealed an association between water availability and the production of different isomers of chlorogenic acids [163]. This knowledge is important in determining the optimal season for plant collection to yield better antiviral efficacy. 

Metabolomics can also be used for studying those metabolic pathways involved in plant-pathogen interaction [159,164,165]. These adapted metabolic pathways can also be determined by analyzing those changes in metabolome during a viral infection. This is important in understanding the mechanism by which plants respond to viral infections and can provide insight into the identification of useful metabolites that may be developed as therapeutic candidates for antiviral intervention. 

A summary of such studies is given in Table 1 below.

## 6. Steps for the Discovery of Antiviral Compounds in Plant Metabolomics Studies

The discovery of compounds with antiviral activities in plants using metabolomics follows a systematic approach, combining different steps and methodologies. The choice of plant selected for metabolomics studies is often predicated upon prior knowledge of the plant’s antiviral properties, either from oral tradition or previous studies. Different plant parts such as stems, roots, leaves, and flowers, are often collected from the wild or from a cultivated environment. Due to the different distribution or constituency of various bioactive compounds in different parts of the plants [178,179,180], a thorough knowledge from literature or indigenous knowledge systems is required to determine the specific plant parts which need to be harvested for the specific application/investigation. 

Post-harvest, sample preparation often involves cleaning, drying, and pulverizing/homogenizing the plant materials, with the subsequent extraction of the bioactive metabolites using various solvents of varying polarities depending on the compound of interest. Phytochemical investigations typically use a solvent mixture for the extraction of both polar and non-polar compounds, which comprises a mixture of alcohols (methanol or ethanol) and water [181,182,183,184,185,186]. Extraction may be followed by sample preparation for NMR, GC-MS, or LC-MS analysis, depending on the aim of the studies and the nature of the targeted metabolites.

Sample preparation for subsequent mass spectrometry analyses is generally more complex than that required for NMR, often requiring prior derivatization of the extracted compounds to improve detection of a wider range of analytes [187]. However, the sensitivity of hyphenated mass spectrometry techniques far surpasses that of NMR. NMR sample preparation generally involves the addition of deuterated methanol (CH3OH-d4), potassium dihydrogen sulfate (which acts as a buffer in a deuterium water (D_2_O) solvent) and trimethyl silane propionic acid (TSP) as an internal reference to detect and correct possible chemical shift during the analyses of multiple samples [188,189]. Data when using either approach are generated in the form of NMR spectra and mass spectrometer chromatograms. 

There are a number of software choices for multivariate data analysis and data processing, including MestReNova [190], MetaboLab [191], the Soft independent modeling by class analogy (SIMCA) [192], Metaboanalyst [193], and Chenomx [194]. Software exclusively designed for mass spectrometry include XCMS (one of the most popular software for MS) [195], MZmine [196], Automated Data Analysis Pipeline (ADAP) [196], Mass Spectrometry-Data Independent Analysis software (MS-DIAL) [197] and METLIN [198].

Multivariate data analysis and data processing are essential steps and precursors to the characterization of bioactive compounds in medicinal plants. The chemical structure of potential antiviral bioactive principles can then be further and more accurately annotated by advanced techniques such as 2D NMR. This is a versatile technique that provides more information about a compound. When used in tandem with relevant databases such as plant metabolome database and reference libraries [199,200], it is helpful in the elucidation of small organic molecules’ structures. 

Subsequently, a bioguided isolation study to determine the antiviral activity of compounds of interest is carried out, increasing the probability of isolating compounds with high bioactive activity [201,202]. A knowledge of natural product chemistry is also invaluable in investigating small organic molecules with medicinal properties from plant sources. Thin-layer chromatography (TLC) and column chromatography are popular choices for compound isolation owing to their convenience and relatively low cost of operation [203]. TLC (or its more sophisticated and improved form, called high-performance thin-layer chromatography) are commonly used techniques that provides a fast and comprehensive overview of most of the compounds present in a plant extract, supports the identification of target compounds in a mixture, and is effective in the isolation of analytes in a sample mixture [204,205]. The basic principle involves the movement of a compound mixture along the mobile phase (solvent), through the stationary phase, where separation occurs depending on the adsorption capacities of the compounds in the mixture. The aforementioned column chromatography (or its highly-improved automated form-high-performance liquid chromatography) is a technique similar to TLC. It works on the same principle, except that instead of the stationary phase on a thin layer in TLC, the solid in the stationary phase is loaded into a long glass column [204,206,207,208].

When determining the antiviral activities of plants, it is important to conduct an assay to evaluate the antiviral potential of the plant of interest to inhibit viral replication, inhibit viral binding to host cell receptors, or interfere with the viral life cycle. An assay commonly used for this purpose is the cytopathic effect inhibition assay (CPE). This involves infecting a host cell culture with the virus, then treating the cells with the plant extract. The cytopathic effect on the cell (cell damage caused by the viral infection) can then be determined [209,210]. Another often-used assay is the plaque reduction assay. Here, an assessment is made of a plant extract’s ability to inhibit viral replication. Cultured cells are seeded to a surface to form a confluent monolayer and infected with a known number of viral particles. The viruses replicate and form plaques while plant extracts are added at different concentrations. Fewer plaques suggest antiviral activity of the plant extract [211,212,213].

Preclinical trials are studies conducted on animal models to evaluate the effect of a test compound or drug for its therapeutic potential [214]. Such trials can be performed in vivo, in vitro, ex vivo, or in silico to gather information regarding the safety and efficacy of the test compound before performing clinical trials on humans [215], which determines the effect of drugs or bioactive compounds on human health outcomes [7,216,217,218]. These medical studies follow specific protocols, including the recruitment of healthy volunteers and patients [219]. With respect to the clinical trials of plant-based antiviral compounds, the trials pass through a number of phases, including initially testing the plant-derived compound/drug on healthy volunteers to determine possible side effects and other safety concerns [220], before testing these on infected or disease patients for drug efficacy.

## 7. Future Directions for the Application of Metabolomics to the Development of Antiviral Therapies from Plant Sources

In the just about 20 years since the introduction of metabolomics [221], some key advancements and significant progress have been made in the field of medicinal plant and natural product research. There remains an immense potential for the full exploration of metabolomics in medicinal plant research and in the search and development of new therapeutic agents. As [222] noted, although many studies have been conducted on plants to determine their effects on viruses, most studies do not isolate and identify active compounds responsible for the antiviral potentials of the tested plants. This is further confirmed by the limited number of studies that reached the level of compound identification and isolation in the last couple of years, as listed in Table 1 above. Some studies reported good plant activities against viruses and even went as far as isolating compounds, but did not elucidate the compound names [177,223]. This presents difficulties in characterizing the bioactive principles responsible for the biological activity of the plant. Taking advantage of advances in metabolomics techniques to identify, isolate, and characterize compounds that have both pharmacological effects and good biological activity will prove beneficial in the development of antiviral leads as part of an effort toward viral eradication. Furthermore, a detailed search of many scientific databases reveals a lack of information on the biosynthetic pathways responsible for producing the plant bioactive compounds. Future studies should harness metabolomics for elucidating the biosynthetic pathways responsible for the formation of identified antiviral metabolites by these plants [224]. This is crucial in optimizing plant cultivation and incorporating genetic engineering strategies for the enhancement of compound synthesis with antiviral bioactivity [221,225].

The emergence of systems biology integrates high throughput data generation and analysis from many platforms to understand complex interactions between different levels of organization in a biological system [226,227]. Omics technology, an indispensable molecular technique in systems biology, incorporates genomics, transcriptomics, proteomics, and metabolomics techniques to foster a better understanding of biological processes [224,228]. The application of a systems biology paradigm in plant studies will provide a more holistic view of the antiviral potentials in medicinal plants. 

## Figures and Tables

**Table 1:** Metabolomics Studies on Antiviral Compounds from Selected Plants.

Plant Species	Compound/ Compound Class	Metabolomics Technique Used	Targeted Virus (es)	Study Outcomes	Reference
*Helichrysum aureonitens*	Chlorogenic acids	UPLC-qTOF-MS	Herpes simplex virus-1 (HSV-1)	Significant reduction of HSV-1 titre values	[163]
*Euphorbia amygdaloides* ssp. *semiperfoliata*	Jatrophene esters (terpenes)	UPLC-MS, ^1^H and ^13^C NMR	Chikungunya virus, HIV-1, and HIV-2	Inhibition of viral replication via activation of protein kinase C	[166]
*Phyllanthus brasiliensis*	Justicidin B (polyphenols)	HPLC and ^13^C NMR	Zika virus	Post-infection intracellular reduction of viral load	[167]
*Lampranthus coccineus* and *Malephora lutea*	Green synthesized silver nanoparticle (AgNPs)	UPLC-MS	HSV-1, HAV-10 virus, and Coxsackie B4	Prevents viral entry into the host cell by binding to the viral envelope glycoproteins	[168]
*Hibiscus sabdariffa*	Protocatechuic acid (hydroxybenzoic acid derivatives)	GC-MS	Human Influenza A Virus	Acid-dependent virus inactivation	[169]
*Elaeodendron croceum*, *Artemisia afra*, and *Adansonia digitata*	13-Hydroxy-9Z,11E-octadecadienoic acid (fatty acid), 13S-Hydroxy-9Z,11E,15Z-octadecatrienoic acid (fatty acids)	NMR	Rift valley fever virus	Interferes with binding of the virion to the cellular receptors, therefore inhibiting viral entry and replication	[108]
*Garcinia cambogia*	Naringin (flavonoid)	LC-HRESIMS	COVID-19	Inhibition of virus replication in the lung cells pre-infection	[170]
*Scaevola spinescens*	Ammarin (phenolic compounds), nodakenetin (psoralens)	HPLC-MS/MS Q-TOF	MS2 bacteriophage	Bacteriophage MS2 plaque reduction	[171]
*Pinellia ternata*	Pinellic acid (long-chain fatty acids)	HPLC	Nasal influenza	Demonstrated potency via activation of antiviral of IgA antibody (Ab) and antiviral IgG1 Ab	[172]
*Ephedra sinica*	4,6-dihydroxyquinoline-2-carboxylic acid, 4-hydroxyquinoline-2-carboxylic acid, and 4-hydroxy-6-methoxyquinoline-2-carboxylic acid (quinoline carboxylic acids)	HPLC-Q-TOF-MS/MS, NMR	COVID-19	Inhibited the infec tivity rate of SARS-CoV-2 S protein-pseudoviruses	[173]
*Rhinacanthus nasutus*	Rhinacanthins C, D, N, Q, and E (naphthoquinones)	NMR, mass spectrometry	Rhinovirus and coxsackievirus	Observed cytopathic effect confirmed antiviral action	[174]
*Bombax ceiba*	Bombasinol A (polyphenols)	NMR	Hepatitis B Virus	Inhibits growth and replication of HepG2 2.2.15 cell lines	[116]
*Phyllanthus urinaria*	Loliolide (benzofurans)	NMR, ESI-LCMS	Hepatitis C Virus (HCV)	Inhibits HCV host cell entry	[175]
*Swietenia macrophylla*	3-hydroxy caruilignan C (polyphenolic compound)	NMR LC-MS	Hepatitis C Virus (HCV)	Inhibits HCV via IFN-stimulated response and IFN-dependent antiviral gene expression	[176]
*Tabernaemontana cymosa*	Coumarin A and B (benzopyrone)	NMR, mass spectrometry	Chikungunya virus (CV)	Inhibits CV host cell infection	[177]

## Data Availability

No research data were generated in this review.
